# Calibration by Proxy

**DOI:** 10.1021/acs.analchem.4c01614

**Published:** 2024-07-09

**Authors:** Willis B. Jones, Abigail J. Crossman, Bradley T. Jones

**Affiliations:** † Department of Chemistry and Biochemistry, University of North Florida, Jacksonville, Florida 32224, United States; ‡ Department of Chemistry, Wake Forest University, Winston-Salem, North Carolina 27109, United States

## Abstract

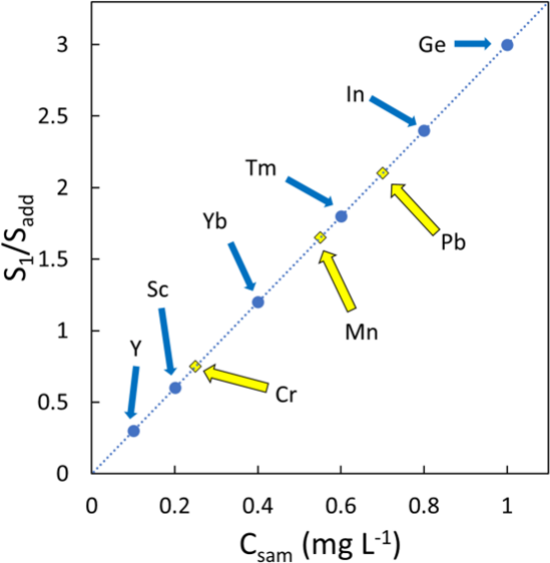

Calibration by Proxy (CbPx) is a matrix-matched calibration method that utilizes multiple internal standard species to build a calibration curve. The technique requires only two solutions: solution 1 containing a sample solution and a suite of internal standards at known concentrations, and solution 2 identical to solution 1, plus an aliquot of a standard containing all analytes and the internal standards at the same concentration. The calibration curve is prepared by plotting the signal measured for each internal standard in solution 1 divided by the signal arising due to the aliquot of internal standard added to solution 2 on the *y*-axis. In this ratio, the sensitivity for each element cancels, because the sample matrix is equal between the solutions. Therefore, the *y*-axis value measured for a specific internal standard is identical to the value that would be measured for any other element present at the same concentrations in the two solutions. Hence, each internal standard serves as a proxy for analyte values. The concentrations of internal standards in solution 1 are plotted on the *x*-axis, and these correspond to any analytes present in solution 1 at the same concentration. CbPx is applied to the analysis of five certified reference materials by inductively coupled plasma optical emission spectrometry (ICP-OES). Percent recoveries for analytes range from 89 to 106%, with relative standard deviations on the order of 1%. A recommended working range for the method is developed through both theoretical simulation and experimental results and then exhibited through the analysis of off-the-shelf vitamin tablets.

Calibration by Proxy (CbPx) is a significant improvement upon the method of standard dilution analysis (SDA), which was first published in this *journal* in 2015.^1,2^ Like SDA, CbPx combines traditional standard additions and internal standard calibration methods, taking the benefits of both while also making the analysis much easier to perform. The SDA technique requires only two solutions for a full calibration: the first solution (solution 1) consists of 50% sample and 50% blank (or pure solvent), while the second solution (solution 2) consists of 50% sample and 50% standard. The standard contains the analytes of interest that are present in the sample and an additional internal standard. The analyte and internal standard signals are measured over time as the solutions mixed dynamically, and a calibration curve is prepared by plotting the analyte signal against the internal standard signal at each point in time. SDA has wide reaching applications as it improves the analysis of samples measured using any instrumental technique that accepts samples in the liquid phase. To date, SDA-type methodologies have been successfully applied to a wide range of sample and analyte types in a variety of analytical methods, including flame atomic emission spectrometry (FAES),^3^ flame atomic absorption spectrometry (FAAS),^4^ microwave-induced plasma optical emission spectrometry (MIP-OES),^5,6^ inductively coupled plasma optical emission spectrometry (ICP-OES),^1,2,7−11^ inductively coupled plasma mass spectrometry (ICP-MS),^12^ Raman spectroscopy,^13^ and visible absorption spectrometry.^1,14^ The novel CbPx method is based on the original SDA idea and maintains the method’s overall simplicity, requiring the preparation of only two solutions to build a full calibration curve.

## Theory

**Figure 1:**
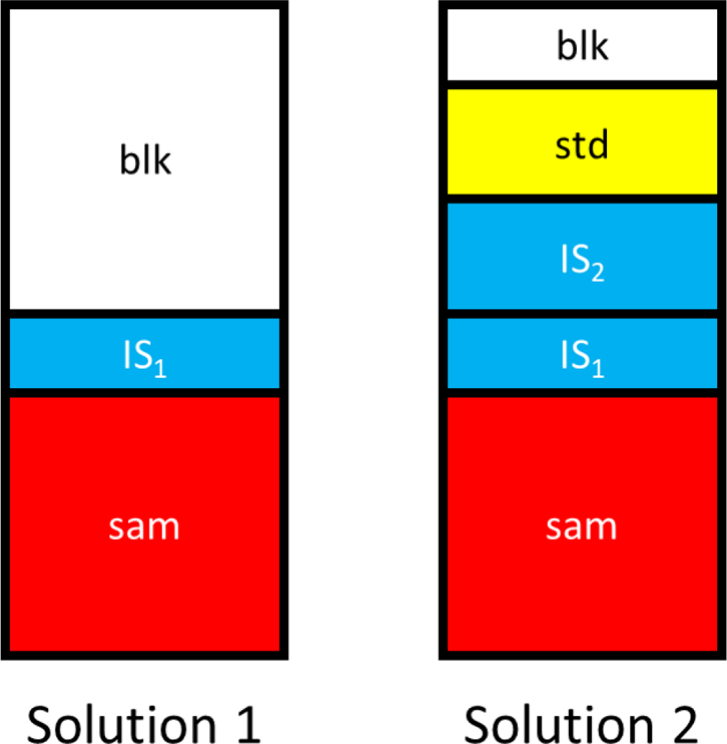
Solution preparation required for Calibration by Proxy.

CbPx requires only two solutions to produce both a full sample analysis and the corresponding calibration curve for each element specific to that sample. Schematic representations of these solutions are presented in Figure 1. Solution 1 contains 50% sample (sam) plus several different internal standard elements present at different known concentrations (IS_1_). Solution 2 contains the same amount of sample as solution 1, the same amount of internal standards as solution 1, plus additional aliquots of a standard (std) containing each analyte of interest, and a solution containing each internal standard (IS_2_). The second solution is prepared in such a way that all analytes and internal standards in the “std” and “IS_2_” portions are present at the same concentration. The remainder of the two solutions consists of blank (blk, pure solvent) to ensure that the solutions have the same final volume and thus a constant sample matrix.

The novelty of CbPx lies in the use of internal standards to produce the calibration curve for every element. The use of multiple internal standards in analytical calibration is not a completely original idea, but the way in which the internal standards in CbPx are employed is. Internal standards in the traditional sense are used to correct for fluctuations within an instrument or in the sample introduction process such as changes in light source intensity or flow rates of carrier gases, among others. In some cases, internal standards are somewhat useful for correcting matrix effects, assuming that the sample matrix influences measured analyte and internal standard signals in a similar fashion. Traditional internal standardization relies on several assumptions: (1) the internal standard is not measurable at detectable levels in the sample itself, (2) it does not spectrally interfere with signals from the analyte, and (3) the internal standard and the analyte react similarly to any changes in the instrumental conditions.^15,16^ If these assumptions are true for a measurement, the use of a signal ratio (analyte to internal standard) to build the calibration curve will correct for instrumental fluctuations.^17^ The success of traditional internal standardization relies on selection of the internal standard itself. This is not always as simple as it seems, especially if more than one analyte is being determined, as the optimal internal standard can vary with analyte species.^18^

Difficulties in the identification of the “ideal” analyte emission wavelength or internal standard species for a given analyte can frequently be minimized through the use of multisignal calibration methods.^19^ Multienergy calibration (MEC) is a matrix-matched calibration technique that requires the preparation of only two solutions for calibration and builds the calibration curve using multiple emission wavelengths for each analyte of interest.^20^ Multi-isotope calibration (MICal) and multispecies calibration (MSC) are similar to MEC in sample preparation but are applicable to only inductively coupled plasma mass spectrometry (ICP-MS). MICal builds a calibration curve using multiple isotopes monitored for each analyte of interest,^21^ while MSC utilizes reaction gases in tandem ICP-MS to form new molecular species, which are then used to construct the calibration curve.^22^ Multi-internal standard calibration (MISC) is the multisignal calibration method that is most similar to CbPx, as it also involves the use of multiple internal standards to build a calibration curve. However, the internal standards in MISC are still used as internal standards in the traditional sense, by taking the ratio of analyte signal to each internal standard used.^23^ All cases of multisignal calibration methods exhibited to date have been shown to reduce calibration error, as the use of multiple signals results in an overall averaging of instrumental noises. Multisignal measurements also reduce the number of calibration solutions required as the calibration curves are generated from multiple species present in an instrument, significantly increasing sample throughput.

In general, the signal (*S*) measured during any analysis is given by the sensitivity for the species being measured (*m*) multiplied by the concentration of the species (*C*). This expression holds for any species in any solution that is to be measured. That is, the signal measured for each internal standard in solution 1 is equal to the sensitivity multiplied by the concentration of each internal standard present in solution 1, as shown in eq 1. The same theory holds for solution 2, with the understanding that the signal arises from two parts: the portions labeled “IS” and “std” in Figure 1, as shown in eq 2.
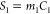
1
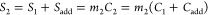
2

The amount of signal corresponding to the amount of additional internal standard added to solution 2 alone is calculated by subtracting the signal from the first solution from the second, as shown in eq 3. The sensitivities *m*_1_ and *m*_2_ are the same, as the two solutions are matrix matched.
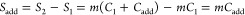
3

The suite of internal standards (IS) chosen for the calibration is not present in the sample; thus, the measured signal for each is directly proportional to the known concentrations of each internal standard in the two prepared solutions. The calibration curve used for sample analysis in CbPx is prepared by plotting a measured signal ratio for each internal standard on the *y-*axis (eq 1 divided by eq 3), with the known internal standard concentrations of solution 1 on the *x-*axis. The result is a straight line as given by eq 4, assuming that the signals measured for all of the internal standards fall within the linear dynamic range of the measurement. The sensitivity for each individual species cancels in the expression, because the two solutions are matrix matched.
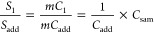
4

**Figure 2:**
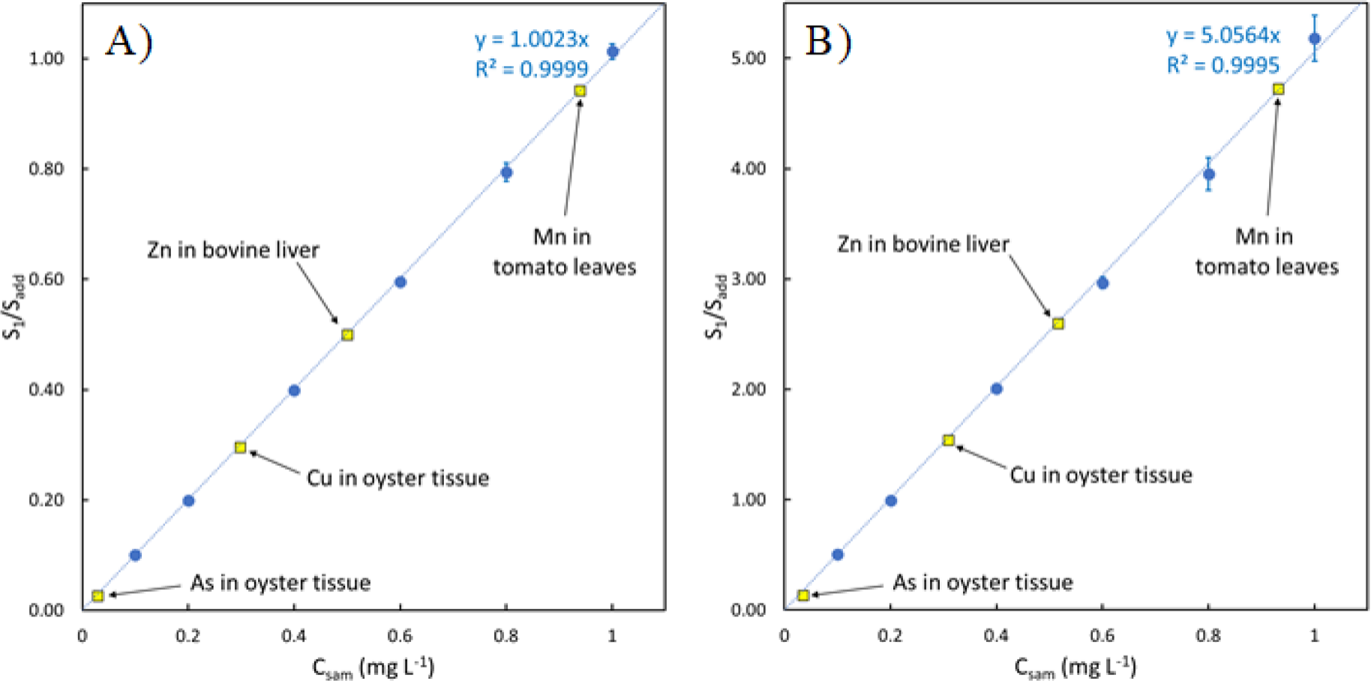
Representative CbPx plots obtained for several analytes in certified reference materials using added concentrations for all species of 1 mg L^–1^ (A) and 200 μg L^–1^ (B).

The calibration curve is prepared by plotting the measured signal ratio *S*_1_/*S*_add_ for each internal standard against the known concentration of each internal standard present in solution 1 (see Figure 2 below). The slope of this plot will be 1/*C*_add_ since the same concentration of each analyte and internal standard is added to solution 2. Also, note that eq 4 holds for any element added since the sensitivities, *m*, cancel for each one. Therefore, the calibration curve prepared with internal standard elements must hold for all analytes, as well. For unknown samples, *C*_1_ in eq 4 is replaced by the unknown concentration (and the desired result) of the analyte in the sample *C*_sam_. Similar to MISC, the use of multiple internal standards minimizes the importance of choosing optimal internal standards, as potential errors introduced through poor selection are minimized through the averaging of results from many species.^23^ Also, since the two solutions are matrix matched, it is not necessary that the internal standards have the same response to the matrix as the analytes. Note that the spacing of the points on the calibration curve is defined by the chosen concentrations of the internal standards in solution 1. Thus, specific points along the curve can be chosen by altering the concentration of the internal standards if desired, tuning the spacing of the points. If desired, the expected concentration of an analyte in the sample can be added to the plot, allowing for an immediate estimate of the relative concentration of an analyte in a sample. This is not possible with other multisignal calibration techniques such as MEC and MISC, as they are limited to given analyte emission wavelengths and internal standard intensities, respectively.

## Materials and Methods

All solutions were prepared using trace metal standards obtained from High-Purity Standards (HPS) (North Charleston, South Carolina, USA). Internal standard stocks were prepared from individual 1000 mg L^–1^ standards for each element. The standard portion of all solutions was prepared from the commercially available HPS “ICP Analytical Mixture 12″, which contains a suite of metals at 100 mg L^–1^.

A detailed description of the solution preparation required for CbPx follows. Each solution contained an identical volume of an unknown sample solution containing analytes at the trace level. The internal standards used to construct the calibration curves for the proof-of-concept measurements presented here were Ge, In, Tm, Yb, Sc, and Y. The concentrations of the internal standards in the aliquot present in both solutions (IS_1_ in Figure 1) were such that the total solution concentrations arising from this portion were 1.0, 0.8, 0.6, 0.4, 0.2, and 0.1 mg L^–1^. The amount of added analytes and internal standards in solution 2 (std and IS_2_ in Figure 1) was such that the concentrations of all species in solution 2 was 1 mg L^–1^ higher than that in solution 1. Thus, the internal standards in solution 2 were present at 2.0, 1.8, 1.6, 1.4, 1.2, and 1.1 mg L^–1^.

All measurements were made using inductively coupled plasma optical emission spectrometry (ICP-OES), but it is important to note that CbPx could be applicable to any analytical technique that measures samples in the liquid phase. An Agilent ICP-OES 5900 (Santa Clara, California, USA) instrument was used for all measurements. The instrument was largely operated under the default conditions. Individual measurements consisted of 10 1 s replicates, and all solutions were measured in triplicate at minimum. Calibration curves were prepared using intensities measured for typical emission wavelengths for each analyte and internal standard (see Tables S1 and S2, Supporting Information).

A suite of certified reference materials was analyzed to validate the CbPx method, including certified reference materials 1568a rice flour, 1566b oyster tissue, 1577b bovine liver, 1573a tomato leaves, and 1547 peach leaves (NIST, Gaithersburg, Maryland, USA). Approximately 0.2 g of each CRM was digested in a solution of 5.0 mL of trace metal grade nitric acid, 2.0 mL of trace analysis grade 30% v/v H_2_O_2_, and 3.0 mL of distilled deionized (DDI) water using an EthosUP high-performance microwave-assisted digestion system (Milestone Inc., Shelton, Connecticut, USA). A digestion “blank” was prepared using the same volumes of reagents but no added solid. The digestion program consisted of a 15 min ramp to 180 °C, a 15 min hold at 180 °C, and a 15 min cooldown. The microwave system remained closed until the temperature inside the digestion vessels dropped to 27 °C. The blank and five CRM solutions were diluted to a final volume of 50 mL with DDI. CbPx measurements were performed using both undiluted digested CRM solution and CRM solution diluted 1:50 as the sample portions of solutions 1 and 2. The expected concentration of each analyte in the digested CRM solutions was calculated using the certified mean concentration values from the CRM certificates and the mass of each CRM digested and diluted to a final volume of 50 mL. The standard portion of solution 2 contained all analytes of interest and the internal standards at a concentration of 1 mg L^–1^.

CbPx measurements were performed in different sample matrices, including DDI, 50% red wine, 50% mouthwash, and 1% (m/v) solutions of both Na and Ca. The 1% Na and Ca solutions were prepared from ACS-grade NaNO_3_ and Ca(NO_3_)_2_, respectively, from Thermo Fisher Scientific (Waltham, Massachusetts, USA). For proof of concept, the sample portion of all solutions was prepared by spiking a known amount of the commercially available HPS “CRDL Detection Limit Standard”, which contains a suite of analyte metals at varying concentrations. Further proof-of-concept measurements were obtained by altering the RF power and nebulizer flow rate instrumental parameters to complement data collected for various sample matrices.

Commercially available off-the-shelf vitamin tablets were analyzed by CbPx to establish a working range for the technique. Three individual tablets were crushed by mortar and pestle and then dissolved in approximately 15 mL of concentrated trace metal grade nitric acid. After the addition of a small amount of DDI, the vitamin tablets were heated gently on a hot plate for several hours, filtered, and diluted to 1 L in volumetric flasks. Solutions 1 and 2 were prepared using each of the three vitamin tablet solutions at three dilution levels (undiluted, 1:10, and 1:100), using standard concentrations of 0.1, 0.5, and 5.0 mg L^–1^. Using the reported mass of metal per tablet from the label, the expected concentrations in the undiluted solution for each tablet were 120 mg L^–1^ of Ca, 0.18 mg L^–1^ of Cr, 2.2 mg L^–1^ of Cu, 110 mg L^–1^ of Mg, 4.2 mg L^–1^ of Mn, 0.090 mg L^–1^ of Mo, 0.117 mg L^–1^ of Se, and 24 mg L^–1^ of Zn.

## Results and Discussion

Validation of the CbPx method was obtained by measuring analyte concentrations in NIST CRMs 1568a rice flour, 1566b oyster tissue, 1577b bovine liver, 1573a tomato leaves, and 1547 peach leaves. Representative CbPx curves are provided in Figure 2 for two different “added” concentrations, 1 and 200 μg L^–1^. The error bars on the internal standard points represent one standard deviation in the signal levels observed for the five different CRM samples. The slope of the calibration curves is equal to 1 divided by the added concentration, which was expected according to eq 4. Note that using different added concentrations does not affect the calculation of the analyte concentration in the sample, as each analyte falls at the same location on the *X*-axis for both curves. Results for all five digested CRMs are presented in Table 1. The expected concentration of each analyte metal in the sample portion of solutions 1 and 2 was calculated by using the certified mean concentration values from the CRM certificates and the mass of each CRM digested and diluted to a final volume of 50 mL. Overall, CbPx provided striking results for all five CRMs. Found analyte recoveries in cases where the expected sample concentration in solution was high were all near 100% with relative standard deviations on the order of 1%. For analytes with expected solution concentrations approaching LOD (31, 10, 14, and 19 μg L^–1^, for As in oyster tissue, Cd in oyster tissue, Mo in bovine liver, and Cu in tomato leaves, respectively), recoveries were still near 100% with slightly higher %RSDs. The results obtained for a wide range of analytes in a variety of certified sample matrices show that CbPx is indeed a powerful analytical calibration technique that requires minimal solution preparation and offers an impressive sample throughput.

**Table 1:** Percent Recoveries Obtained for Analytes in Certified Reference Materials^a^

	**rice flour**		**oyster tissue**		**bovine liver**		**tomato leaves**		**peach leaves**	
**element**	**recovery (%)**	**st dev (%)**	**recovery (%)**	**st dev (%)**	**recovery (%)**	**st dev (%)**	**recovery (%)**	**st dev (%)**	**recovery (%)**	**st dev (%)**
**As**			92	9						
**Ba**			92^c^	1			91.6^c^	0.3	94.1	0.4
**Ca**			101.5^b^	0.7	108	1	90^b^	3	100^b^	3
**Cd**			105	5						
**Cu**			98	1	104.1	0.6	97	5		
**Fe**	106	2	99	1	104	1	93	1	88.8	0.8
**Mg**	94	2	96.7^b^	0.7	101.0^b^	0.9	87.3	0.8	94^b^	1
**Mn**	92.3	0.8	100.0	0.9	103	2	94.8	0.8	91.5	0.5
**Mo**					100	12				
**Sr**			95^c^	3			96^c^	1	98.7	0.9
**Zn**	91	1	100.0^b^	0.5	103.1	0.6	93	1	90	1

a Recoveries and standard deviations are the result of triplicate measurements using three separate emission lines for each analyte.

b Sample was diluted 1:50 for analysis.

c Concentration listed on CRM certificate, but not certified.

Analyte limits of detection for CbPx were determined by preparing solutions 1 and 2 in DDI without any added sample. The concentration of the standard in solution 2 was 1 mg L^–1^ for all analytes and internal standards. Solutions 1 and 2 were both measured 10 times, and CbPx curves were constructed for each measurement. The limits of detection reported in Table 2 were calculated as three times the standard deviation of the “blank” concentrations found using each of the 10 measurements. LODs for CbPx using ICP-OES are similar to those obtained through traditional ICP-OES measurements, on the order of single digits μg L^–1^.

**Table 2:** Analyte Limits of Detection Obtained from 10 Determinations of a Sample Blank

**element**	**LOD(μg L**^**–1**^**)**	**element**	**LOD(μg L**^**–1**^**)**
As	5	Mo	3
Be	2	Ni	2
Ca	7	Pb	5
Cd	2	Sb	5
Co	2	Se	5
Cr	2	Tl	8
Cu	2	U	10
Mg	2	V	2
Mn	2	Zn	2

**Figure 3:**
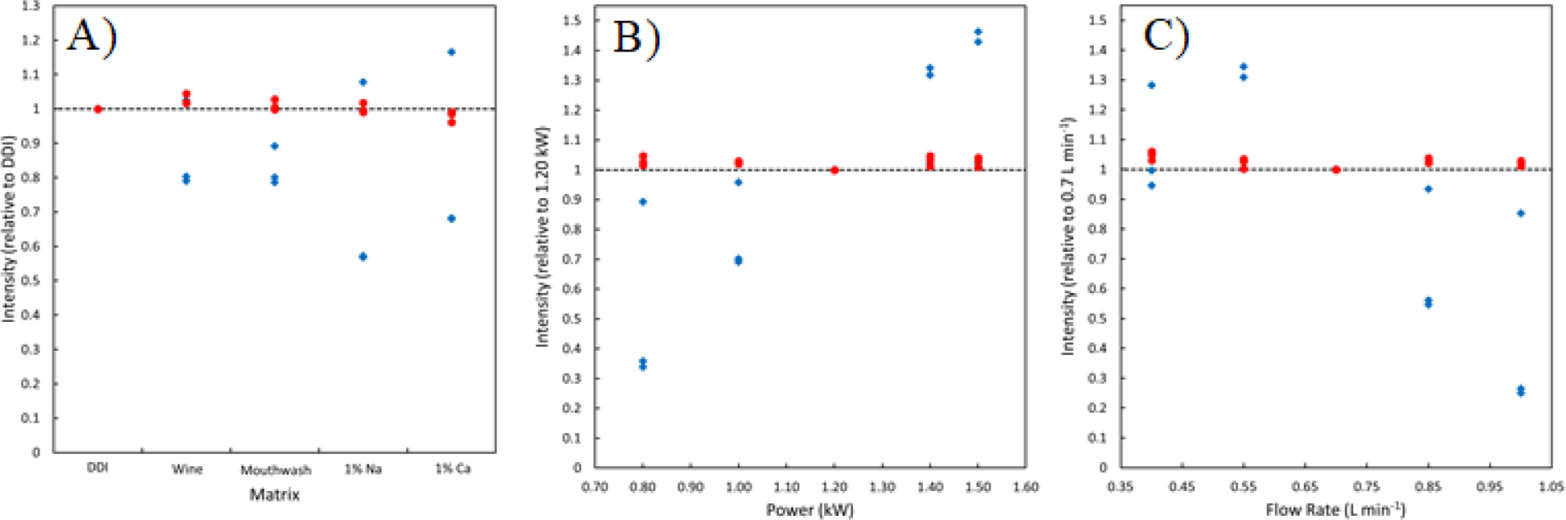
Raw intensities (blue diamonds) vs intensity ratio (red circles) comparison obtained for three Cr emission lines when changing sample matrix (A), plasma power (B), and nebulizer flow rate (C). Intensities are relative to DDI for the matrix comparison, and relative to default instrument conditions for both plasma power and nebulizer flow rate, represented by the dashed black line.

As both solutions 1 and 2 contain equal amounts of sample, CbPx is a matrix-matched calibration technique, correcting for sample matrix effects. In the simplest terms, a sample matrix (everything that is present in a sample that is not analyte) can drastically affect any measured signals. A visual representation of matrix effects on Cr emission intensity is provided for different sample types and plasma conditions in Figure 3. Raw Cr signals for three monitored emission lines measured in solution 1 are colored blue, with signal ratios (*S*_1_ divided by *S*_add_) shown in red. All intensities are relative to DDI (the cleanest matrix) when comparing sample matrices, and relative to the default plasma parameters. Raw emission intensities can be affected by sample matrix and plasma conditions in different ways, with a significant signal depression observed in a 1% Ca solution for two of the Cr lines (over 40%), with the other line showing a 20% enhancement, for example. However, using the signal ratios to build calibration curves results in a correction for any signal changes, as each emission line is ratioed to itself between the two solutions. This is evident from the red data in Figure 3, which shows the measured signal ratio for all matrices and plasma conditions largely unchanged relative to DDI and default plasma parameters, even for cases in which the raw measured signals for an analyte line change drastically.

Proof of the matrix matching concept for CbPx was obtained by spiking known amounts of a suite of analyte metals into a variety of sample matrices, including DDI, 50% v/v red wine, 50% v/v mouthwash, and 1% m/v solutions of both Na and Ca. Note that even for sample matrices that are known to exhibit severe matrix effects such as mouthwash and high calcium,^1,2,7^ the use of matrix-matched solutions results in measured signal ratios that are identical for all analytes across all sample matrices. A summary of nine analyte metals is provided in Table 3. Recoveries and standard deviations are the result of triplicate measurements using three separate emission lines for each analyte. For each sample matrix tested as proof of concept, percent recoveries for each analyte are near 100% with a standard deviation on the order of 1%. The standard concentration for all measurements was 1 mg L^–1^. The combined recovery for all analytes across all measured sample matrices (*n* = 135) was 98%, with an RSD of 3%.

**Table 3:** Percent Recoveries for a Suite of Analyte Metals Spiked into Various Sample Matrices^a^

	**spike**	**DDI**		**wine**		**mouthwash**		**1% Na**		**1% Ca**	
**element**	(mg L^–1^)	**recovery (%)**	**st dev (%)**	**recovery (%)**	**st dev (%)**	**recovery (%)**	**st dev (%)**	**recovery (%)**	**st dev (%)**	**recovery (%)**	**st dev (%)**
**Be**	0.10	98	1	97	1	97	1	102	2	97.6	0.6
**Cd**	0.10	96	4	94	2	100	4	102	5	95	4
**Co**	1.00	102.2	0.8	96.3	0.7	97	2	99	3	96	1
**Cr**	0.20	99	2	102	2	99	2	102	2	97	2
**Cu**	0.50	102	3	95	3	97	5	100	3	95	3
**Mn**	0.30	98.4	0.6	107	7	97.1	0.8	102	2	98.1	0.6
**Ni**	0.80	102	2	97	4	96	3	100	4	95	2
**V**	1.00	97	1	98	1	95.6	0.9	96	2	97	1
**Zn**	0.40	102	3	97	3	100	5	105	5	98	1

a Recoveries and standard deviations are the result of triplicate measurements using three separate emission lines for each analyte.

Further proof of concept of the correctional power of the CbPx method was obtained by spiking known amounts of the same analyte metals into DDI and altering instrumental operation parameters, including the RF power and nebulizer flow rate. These plasma conditions can significantly alter analytical signals measured, as shown in Figure 3, with Cr emission intensities for two of the monitored emission wavelengths depressed by approximately 70%, for example. Summaries for the selected analytes are listed in Tables 4 and 5. Even when altering the instrumental operating parameters to extreme conditions, the percent recoveries for each analyte are near 100% with a standard deviation on the order of 1%. The combined recovery for all analytes across all measured RF powers (*n* = 135) was 98%, with an RSD of 3%. The combined recovery for all analytes across all measured nebulizer flow rates (*n* = 135) was 98%, with an RSD of 3%. Such significant changes to plasma operational parameters lead to markedly different plasma robustness, which is typically not easily correctable using traditional calibration techniques.

**Table 4:** Percent Recoveries for a Suite of Analyte Metals Spiked into DDI when Altering the Plasma RF Power^a^

	**spike**	0.8 kW		1.0 kW		1.2 kW		1.4 kW		1.5 kW	
**element**	(mg L^–1^)	**recovery (%)**	**st dev (%)**	**recovery (%)**	**st dev (%)**	**recovery (%)**	**st dev (%)**	**recovery (%)**	**st dev (%)**	**recovery (%)**	**st dev (%)**
**Be**	0.10	99	2	99	2	96.8	0.9	96	2	97	1
**Cd**	0.10	98	3	98	2	94	2	96	2	95	2
**Co**	1.00	100	2	100	1	100	2	101.6	0.8	100	1
**Cr**	0.20	99	2	99	3	96	3	100	2	99	2
**Cu**	0.50	102	4	100	3	100	3	102	2	101	2
**Mn**	0.30	98	1	98.1	0.9	96.4	0.5	99	1	98	1
**Ni**	0.80	104	5	99	2	99	2	102	2	100	2
**V**	1.00	95.1	0.9	94.2	0.9	93	1	96	1	96.1	0.7
**Zn**	0.40	97	12	99	3	100	3	99	1	99	2

a Recoveries and standard deviations are the result of triplicate measurements using three separate emission lines for each analyte. The default plasma RF power is 1.2 kW.

**Table 5:** Percent Recoveries for a Suite of Analyte Metals Spiked into DDI when Altering the Nebulizer Flow Rate^a^

	**spike**	**0.40** L min^–1^		**0.55** L min^–1^		**0.70** L min^–1^		**0.85** L min^–1^		**1.00 L** min^–1^	
**element**	(mg L^–1^)	**recovery (%)**	**st dev (%)**	**recovery (%)**	**st dev (%)**	**recovery (%)**	**st dev (%)**	**recovery (%)**	**st dev (%)**	**recovery (%)**	**st dev (%)**
**Be**	0.10	96	1	98	2	96.8	0.9	99	1	100	2
**Cd**	0.10	94.4	2	97	2	94	2	98	3	103	5
**Co**	1.00	98.6	0.7	100	1	100	2	100	2	99	3
**Cr**	0.20	99	2	99	2	96	3	100	3	99	3
**Cu**	0.50	100	2	101	3	100	3	101	2	100	4
**Mn**	0.30	98.5	0.9	98	1	96.4	0.5	98	1	98	1
**Ni**	0.80	98	2	100	2	99	2	101	2	102	4
**V**	1.00	95	1	94	1	93	1	95.4	0.9	95	1
**Zn**	0.40	97.1	0.6	100	2	100	3	103	7	100	8

a Recoveries and standard deviations are the result of triplicate measurements using three separate emission lines for each analyte. The default nebulizer flow rate is 0.70 L min^–1^.

**Figure 4:**
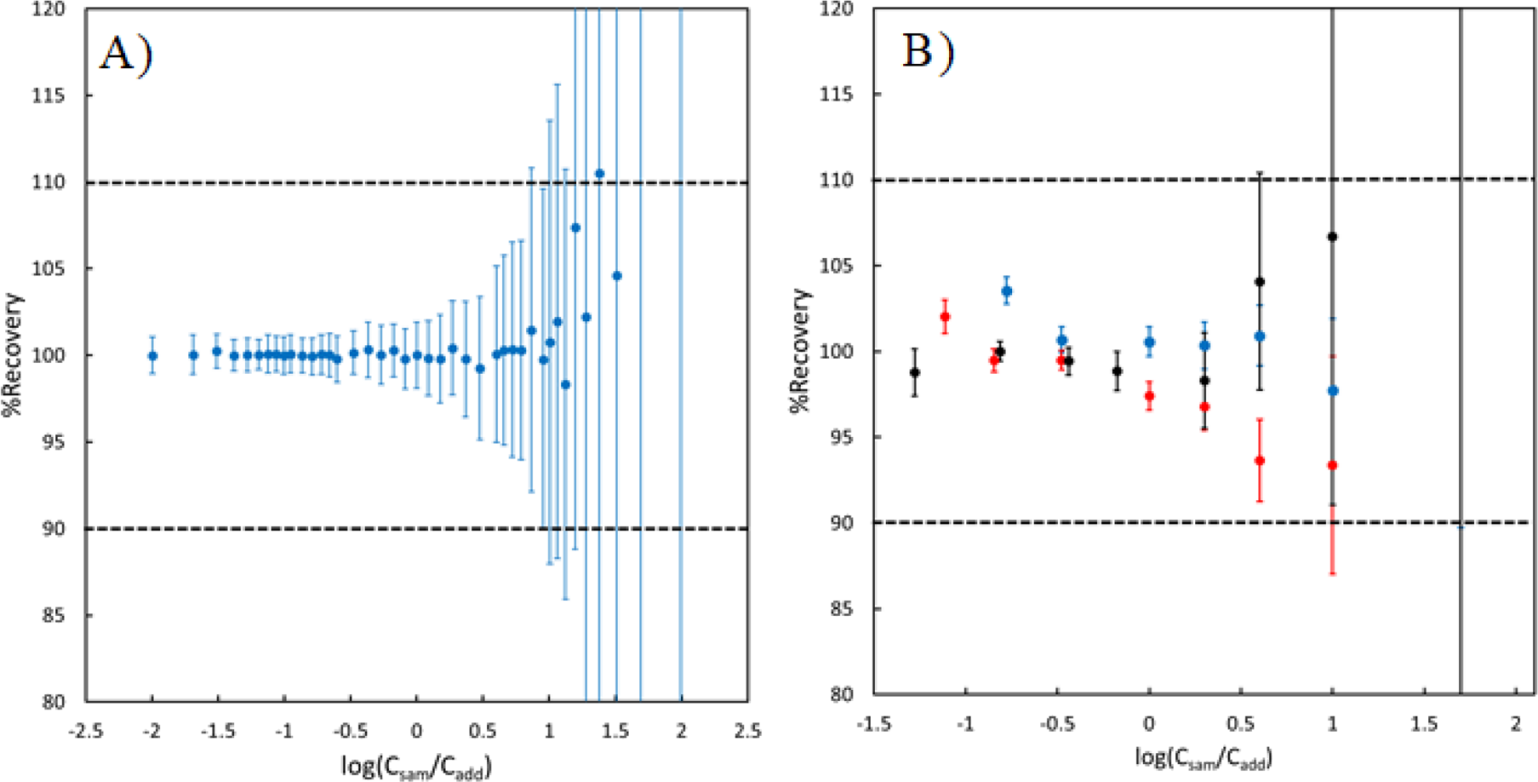
Recovery values using both simulated signals (A) and real signals for Cd measured using different samples and standard concentrations (B). The blue, red, and black points for the real data correspond to sample concentrations of 1 mg L^–1^, 500 μg L^–1^, and 200 μg L^–1^, respectively. Dashed horizontal lines correspond to the recovery range of 90–110%.

Looking at eqs 3 and 4, it is evident that the calculation of analyte concentration in the sample will fail when the signal measured for an analyte in solution 2 approaches that of solution 1. This occurs when the concentration of the analyte in the sample is much higher than the added concentration of the analyte in the standard. This was exhibited by simulating signals, and then the simulated data set was used to calculate the concentrations of analyte in the “sample”. Ratios *S*_1_/*S*_add_ of desired magnitudes were simulated in sets of 100, with RSDs of 1%. The “true” sample concentration was calculated by using eq 4, using the desired ratios. “Experimental” sample concentrations were calculated by using the 100 simulated ratios for each desired ratio. A theoretical %recovery was calculated for each simulated ratio as the experimental concentration divided by the true concentration times 100. The simulated data are compiled in Figure 4A, which shows both the average and standard deviation of the 100 simulated %recoveries when changing the concentration of analytes in the sample relative to the standard. For low ratios (when the concentration in the sample is less than the standard), the theoretical recovery is near 100%, with a standard deviation across the 100 simulated ratios on the order of 1%. The recovery stays near 100% as the sample concentration increases, but the error in the recovery slowly increases. From a purely theoretical standpoint, obtained recoveries are unsatisfactory when the concentration in the sample is more than 10 times the concentration added from the standard (log(*C*_sam_/*C*_add_) > 1), with standard deviations on the order of 10% at minimum.

The theoretical breakdown of the calculation is complemented by an experimental breakdown observed in real, prepared solutions of known concentration. Solution 1 was prepared using three concentrations of analytes: 1 mg L^–1^, 500 μg L^–1^, and 200 μg L^–1^. A series of solution 2 was prepared using different analyte concentrations ranging from 204 μg L^–1^ to 7 mg L^–1^. All solutions were prepared in a sample matrix of DDI. Analyte and internal standard signals in each solution were measured in triplicate using ICP-OES, and signal ratios and “added” concentrations for different combinations of solutions 1 and 2 were calculated. For example, when using solution 1 containing 200 μg L^–1^ of analyte, the sample concentration in each prepared solution 2 is defined as 200 μg L^–1^. Thus, for solution 2 that contains 204 μg L^–1^of the analytes, the sample concentration is 200 μg L^–1^and the added concentration is 4 μg L^–1^ (204–200 = 4). Concentrations of the analyte in the “sample” portion of the solution were calculated using the prepared CbPx curves according to eq 4 and the added standard concentration. The results of the possible combinations of solutions 1 and 2 using Cd as the analyte are presented in Figure 4B. The experimental and theoretical recoveries follow a similar trend.

When the added standard concentration is larger than the sample concentration, the experimental recoveries are near 100%. As the ratio increases, the error in the recovery also increases, to the point where the results are analytically unreliable, with standard deviations above 10% when the concentration in the sample is >10 times the concentration added from the standard. The increasing error between measurements is exaggerated further as the analyte concentration in the sample decreases. The standard deviation of the recoveries when using a sample concentration of 200 μg L^–1^ (the black points in the plot) is larger across all experimental ratios when compared to higher sample concentrations. The trends observed in the results for Cd in Figure 4B were the same for every other analyte that was measured. When using CbPx for analysis of real samples, it is suggested that the added standard concentration be kept >10% of the sample concentration if possible, especially if the concentration of analytes in the sample is approaching the limit of detection for the instrumental suite in use. As is the case with any calibration method, some baseline knowledge of the expected analyte concentrations is required.

However, the results presented in Figure 4 exhibit a particular strength of the CbPx method. If all analyte concentrations are expected to be on the order of 1 mg L^–1^, using an added standard concentration of 1 mg L^–1^ provides reliable analytical recoveries for all analytes ranging from the method’s limit of detection (single digit μg L^–1^) to 10 mg L^–1^, a range of four orders of magnitude. If it is found after the initial analysis that a desired analyte has a concentration higher than the upper limit of the range, the sample is simply diluted and solutions 1 and 2 are prepared again and measured separately, as would be the case for any calibration technique.

Table 6 presents the average recoveries obtained for three individually digested vitamin tablets at three different dilution levels and three standard levels, resulting in nine combinations of sample and standard levels. As expected, metals present in high concentrations (Ca and Mg) are accurately determined only when the tablet solutions are significantly diluted to a point where the sample and standard concentrations are on similar orders, providing adequate results for only three of the nine possible combinations.

**Table 6:** Percent Recoveries Obtained for the Measurement of Metals in Vitamin Tablets at Three Sample Dilution Levels and Three Standard Concentrations

**element**	**undiluted**(mg L^–1^)	*n* (out of 9)	**recovery (%)**	**st dev (%)**
**Ca**	120	3	108	6
**Cr**	0.18	6	119	7
**Cu**	2.2	7	113	5
**Mg**	110	3	114	7
**Mn**	4.2	7	110	5
**Mo**	0.09	3	113	5
**Se**	0.117	3	125	6
**Zn**	24	6	108	7

Similarly, metals present at low concentrations (Mo and Se) are accurately determined only when the tablet solution was undiluted, as any dilution leads to solution concentrations approaching LOD. Other metals present in the tablet were successfully determined in more combinations of added standard concentration and sample dilution level.

Typically, successful analytical calibration requires the preparation and measurement of a series of standard and sample solutions. One might look at a method such as CbPx and think at first glance that the method is only a “two-point” calibration, which is largely considered insufficient. However, CbPx is in fact akin to a traditional multipoint calibration method. Although only two solutions are prepared and measured individually, each solution contains six internal standards that are used to construct the calibration curve and each of the internal standard concentrations is prepared individually from single element solutions. The use of the previously described signal ratios allows for the internal standards to serve as proxies for all analytes, significantly simplifying the analysis process as six individual standard ratios are obtained even though only two static solutions are analyzed. The authors have published elsewhere that SDA-type methodologies offer impressive, repeatable analytical results even when using only a two-point calibration.^24^ In addition, the authors have published full comparisons of SDA-derived techniques to traditional calibration methods, and CbPx offers similar results at worst (and significantly improved results when compared to difficult sample matrices and plasma parameters) when compared to traditional internal standard calibrations. CbPx offers percent recoveries of approximately 100% with relative standard deviations on the order of 1% for all measured analytes regardless of the sample matrix or alterations of plasma conditions. Traditional internal calibrations rarely correct for severe matrix effects, with measured recoveries ranging from approximately 50 to 200%, with relative standard deviations frequently on the order of 10% or higher.^1,2,7,24^

In general, the same limitations apply to CbPx that apply to any successful analytical calibration: the concentration of the sample and the standard should not be vastly different, and the concentration in the sample must be above the LOD for successful determination. Recoveries for each analyte in the tablets ranged between 110 and 120%, slightly elevated from the values reported on the sample label. Further validation using traditional external standard calibration and standard dilution analysis returned similarly elevated concentrations with analyte recoveries of approximately 120%. Thus, it is more likely than not that the concentration of trace metals in the vitamin tablets was indeed higher than the value reported on the bottle’s label. These results affirm that the added standard concentration should be >10% of the sample concentration. This working range can be achieved either by decreasing the sample concentration in both solutions through dilution or by increasing the concentration of the standard portion of solution 2.

## Conclusions

CbPx has proven to be a simple, fast, and accurate analytical calibration method. Benefits of the CbPx method include (1) matrix effect correction through matrix matching, (2) correction of internal instrumental fluctuations such as plasma power and flow rates, (3) high sample throughput due to minimal solution preparation, and (4) the use of a single calibration curve to determine any analytes present in a given sample. CbPx requires the preparation of only two solutions to perform a full analytical calibration for each individual sample. The first solution contains a portion of the sample solution and a suite of internal standards, and the second solution contains the same sample solution, the same internal standards, and a standard consisting of all analytes of interest and the internal standards, all at the same concentration. A calibration curve is prepared by plotting a signal ratio of the internal standard signals (signal from solution 1 divided by the signal from the added portion of solution 2) on the *y*-axis, with their known, prepared concentration in solution 1 on the *x*-axis. The use of a signal ratio to build the calibration curve, as well as the addition of an identical amount of all analytes and internal standards to solution 2, allows for the internal standards to serve as proxies for the analyte values. The concentration of any analytes of interest is calculated from the same measured analyte signal ratio and the known concentration of the added analyte standard in the second solution. The proof of concept for CbPx was obtained using ICP-OES, but the method could be applied to any multielement simultaneous instrumental technique that measures samples in the liquid phase. In addition, CbPx should be applicable to any instrumental technique that measures static sample solutions regardless of simultaneous detection capabilities. No additional hardware for successful CbPx is required as the instrument simply measures signal levels of a suite of analytes and internal standards in two separate, static, prepared solutions.

The proof-of-concept CbPx measurements presented took aim at three targets: (1) proof of matrix effect correction, (2) establishment of a working range for the technique, and (3) validation of the method through certified reference materials. CbPx was successful in accomplishing all three of these benchmarks. CbPx was shown to correct for matrix effects, even for extreme cases, such as high salt concentrations and severely altered plasma measurement conditions. Percent recoveries for all analytes under all testing conditions fell in the range of 94–107%, with relative standard deviations on the order of 5% or lower. CbPx as a method was validated through the measurement of certified reference materials, with percent recoveries for all analytes measured across five CRMs falling in the range of 89 to 106%, with relative standard deviations on the order of 1%.

## Supporting Information Available

Experimental details, including instrumental operational parameters and monitored species emission wavelengths (PDF)
