# Supplementary material for: Calibration by Proxy
Source: Anal Chem. 2024 Jul 9;96(29):11906–14. doi: 10.1021/acs.analchem.4c01614 (PMC11270529; doi:10.1021/acs.analchem.4c01614)
Supplement: Supplementary file 1 — ac4c01614_si_001.pdf [file ac4c01614_si_001.pdf]

Supporting Information

Calibration by Proxy

Willis B. Jones[1*], Abigail J. Crossman[1], and Bradley T. Jones[2]

[1] Department of Chemistry and Biochemistry, University of North Florida, Jacksonville, FL 32224

[2] Department of Chemistry, Wake Forest University, Winston-Salem, NC 27109

* Corresponding author. Email: w.jones@unf.edu

Supporting Information

Table S1. Parameters used for operation of the ICP-OES.

Parameter	Value
RF Power	1.2 kW
Nebulizer Flow Rate	0.70 L min^{-1}
Plasma Flow Rate	12.0 L min^{-1}
Auxiliary Flow Rate	1.00 L min^{-1}
Pump Speed	12 rpm
Uptake Delay	25 s
Stabilization Time	15 s
Rinse Time	30 s

Table S2. Selected emission wavelengths for all analytes and internal standards.

Element	Function	Wavelength (nm)				
As	Analyte	188.980	193.696	197.198		
Ba	Analyte	233.527	455.403	493.408		
Be	Analyte	234.861	313.042	313.107		
Ca	Analyte	393.366	396.847	422.673		
Cd	Analyte	214.439	226.502	228.802		
Co	Analyte	228.615	230.786	237.863		
Cr	Analyte	267.716	283.563	357.868		
Cu	Analyte	219.959	223.009	327.395		
Fe	Analyte	234.350	238.204	239.563	259.940	
Ge	Internal Standard	206.866	219.871	259.253	265.117	270.962
In	Internal Standard	230.606	325.609	410.176		
Mg	Analyte	279.553	280.270	258.213		
Mn	Analyte	257.610	259.372	294.921		
Mo	Analyte	202.032	204.598	277.539		
Ni	Analyte	216.555	222.486	230.299		
Pb	Analyte	220.353	283.305	405.781		
Sb	Analyte	206.834	217.582	231.146		
Sc	Internal Standard	335.372	337.215	357.634	361.383	364.278
Se	Analyte	196.026	203.985			
Sr	Analyte	216.596	407.771	421.552	460.733	
Tl	Analyte	276.789	351.923			
Tm	Internal Standard	342.508	370.026	379.576	384.802	
U	Analyte	367.007	385.957	409.013		
V	Analyte	292.401	292.464	311.837		
Y	Internal Standard	324.228	332.788	358.452	360.074	371.029
Yb	Internal Standard	212.674	222.447	289.138	369.419	398.799
Zn	Analyte	202.548	206.200	213.857		
